# Childhood craniopharyngioma: greater hypothalamic involvement before surgery is associated with higher homeostasis model insulin resistance index

**DOI:** 10.1186/1471-2431-9-24

**Published:** 2009-04-02

**Authors:** Christine Trivin, Kanetee Busiah, Nizar Mahlaoui, Christophe Recasens, Jean-Claude Souberbielle, Michel Zerah, Christian Sainte-Rose, Raja Brauner

**Affiliations:** 1 AP-HP, Hôpital Necker-Enfants Malades, Service d'Explorations Fonctionnelles, Paris, 75743, France; 2 Université Paris Descartes and AP-HP, Hôpital Bicêtre, Unité d'Endocrinologie Pédiatrique, Le Kremlin Bicêtre, 94275, France; 3 AP-HP, Hôpital Albert Chenevier, Service de Psychiatrie, Créteil, 94000, France; 4 Université Paris Descartes and AP-HP, Hôpital Necker-Enfants Malades, Service de Neurochirurgie Pédiatrique, Paris, 75743, France

## Abstract

**Background:**

Obesity seems to be linked to the hypothalamic involvement in craniopharyngioma. We evaluated the pre-surgery relationship between the degree of this involvement on magnetic resonance imaging and insulin resistance, as evaluated by the homeostasis model insulin resistance index (HOMA). As insulin-like growth factor 1, leptin, soluble leptin receptor (sOB-R) and ghrelin may also be involved, we compared their plasma concentrations and their link to weight change.

**Methods:**

27 children with craniopharyngioma were classified as either grade 0 (n = 7, no hypothalamic involvement), grade 1 (n = 8, compression without involvement), or grade 2 (n = 12, severe involvement).

**Results:**

Despite having similar body mass indexes (BMI), the grade 2 patients had higher glucose, insulin and HOMA before surgery than the grade 0 (P = 0.02, <0.05 and 0.02 respectively) and 1 patients (P < 0.02 and <0.03 for both insulin and HOMA). The grade 0 (5.8 ± 4.9) and 1 (7.2 ± 5.3) patients gained significantly less weight (kg) during the year after surgery than did the grade 2 (16.3 ± 7.4) patients. The pre-surgery HOMA was positively correlated with these weight changes (P < 0.03).

The data for the whole population before and 6–18 months after surgery showed increases in BMI (P < 0.0001), insulin (P < 0.005), and leptin (P = 0.0005), and decreases in sOB-R (P < 0.04) and ghrelin (P < 0.03).

**Conclusion:**

The hypothalamic involvement by the craniopharyngioma before surgery seems to determine the degree of insulin resistance, regardless of the BMI. The pre-surgery HOMA values were correlated with the post-surgery weight gain. This suggests that obesity should be prevented by reducing inn secretion in those cases with hypothalamic involvement.

## Background

Obesity is a major problem in some children with hypothalamic-pituitary lesions. The risk factors for obesity in children surviving brain tumors are the hypothalamic location, tumor histology, particularly craniopharyngioma and pilocytic astrocytoma, and the extent of surgery [1]. Long-term survivors of childhood cancer are at increased risk of suffering from metabolic syndrome [2,3] and nonalcoholic fatty liver disease [4]. There is, presently, no effective long-term treatment [5,6].

The exact cause of this obesity is unclear. Lesions of the ventromedial hypothalamus produce obesity and hyperinsulinemia in rats [7], while complete vagotomy reverses the obesity and lowers plasma insulin [8]. Two explanations have been advanced. One postulates that the damage leads to hyperphagia and obesity via a direct neural mechanism involving the central appetite centers and that the hyperinsulinemia is secondary to the obesity. The other postulates that the damage causes disinhibition of vagal tone at the pancreatic β-cells, which leads to insulin hypersecretion and obesity.

There is a link between the degree of hypothalamic involvement evaluated by magnetic resonance imaging (MRI) and the weight of a child before and after surgery to remove the craniopharyngioma [9-12], but exactly how this leads to obesity is unknown. The destruction or functional impairment of the hypothalamus is probably responsible for the failure to integrate neuronal, hormonal and metabolic signals from the body, leading to changed feeding behavior. Insulin-like growth factor (IGF) 1, insulin, leptin and ghrelin may all be involved. We have shown that the pre-surgery plasma fasting insulin concentrations of children with craniopharyngioma are positively correlated with the weight change (kg) during the year after surgery [13]. Others [14,15] have suggested that the higher-than-expected plasma leptin concentration for the body mass index (BMI) indicates a disturbed feedback control of leptin secretion due to the craniopharyngioma and/or surgery. The fasting plasma ghrelin concentrations of adults [16] and children [17] operated on for craniopharyngioma are not different from those of obese controls. Both authors suggest that an elevated fasting ghrelin is unlikely to be responsible for the obesity that occurs after hypothalamic damage.

### Objective

To evaluate the pre-surgery relationship between the degree of hypothalamic involvement by a craniopharyngioma, determined by MRI, and the insulin resistance, evaluated by the homeostasis model insulin resistance index (HOMA), in a population of prepubertal children. We also measured the plasma concentrations of IGF-1, leptin, soluble leptin receptor (sOB-R) and ghrelin, before and 6–18 months after surgery, when the blood sample was large enough. We compared these plasma concentrations between them and to the changes in body weight.

## Methods

### Patients

A series of 27 patients (15 boys) were selected consecutively from a cohort of craniopharyngiomas operated on at the Hôpital Necker-Enfants Malades. The selection criteria were a complete surgical history at the same department, follow-up performed by the same pediatric endocrinologist (R Brauner) and the availability of at least one pre-surgery plasma sample.

The pre-surgery neuroradiological data were reviewed retrospectively by one author (C Sainte-Rose) blinded to the clinical data. The relationship between the hypothalamus and the craniopharyngioma was used to assign patients to a grade: grade 0 included those without hypothalamic involvement (n = 7); grade 1 included those who had an identifiable hypothalamus that was compressed and displaced by a suprasellar or intraventricular tumor (n = 8); and grade 2 included those who had severe hypothalamic involvement (n = 12) [10].

All the patients were aged 8.3 ± 3.5 (2.8–15.7) years at the first evaluation before surgery and were followed for at least one year after surgery.

### Protocol

Informed consent for the evaluations and treatments was obtained from the children's parents. The Ethical Review Committee (Comité de Protection des Personnes Ile de France III) stated that "this research was found to conform to generally accepted scientific principles and research ethical standards and to be in conformity with the laws and regulations of the country in which the research experiment was performed".

The endocrine status of each patient was evaluated at 8:00 a.m in a fasting state both before surgery and after surgical resection of craniopharyngioma, when the patients were on replacement thyroxin, hydrocortisone and desmospressin. Before surgery, 81% of the patients lacked growth hormone (GH), 37% lacked thyroid stimulating hormone, 22% lacked adrenocorticotropin hormone, and 7% lacked vasopressin. The frequency of deficiencies did not vary with the degree of hypothalamic involvement. Replacement thyroxin and hydrocortisone were always initiated the day before surgery; the plasma cortisol concentrations of the patients with normal plasma cortisol concentrations before surgery were measured at 8:00 a.m. after surgery, 12 hours after the last dose of hydrocortisone just before leaving the hospital. All, except one, had GH, thyroid stimulating hormone, adrenocorticotropin hormone and vasopressin deficiencies after surgery. At the last clinical evaluation, five were of prepubertal age, two had spontaneous pubertal development and the 20 others had complete gonadotropin deficiency.

The fasting plasma concentrations of at least one of insulin, leptin, sOB-R, and ghrelin and calculation of HOMA and free leptin index (FLI) were measured before surgery. The interval between surgery and the second evaluation of these concentrations was 6 to 18 months, depending on sample availability. All patients remained prepubertal and none of them was given GH or sex steroids during the study period.

### Methods

The endocrine evaluation included the response to a GH stimulation test, the fasting plasma concentrations of glucose, insulin, IGF-1, free thyroxin, cortisol, and concomitant plasma and urinary osmolalities. These parameters were also evaluated after surgery.

The GH secretion was first assessed by the sequential arginine-insulin test and then by the ornithin test. The insulin test was not performed in patients in a critical medical state. GH deficiency was defined by a GH peak of less than 10 μg/L. The hypothalamic-pituitary-thyroid axis was assessed by measuring plasma free thyroxin concentrations, and any deficiency (< 12 pmol/L) was treated with thyroxin (75–100 μg/m^2^/day). The hypothalamic-pituitary-adrenal axis was assessed by measuring the plasma cortisol concentration at 8:00 a.m., and any deficiency (<80 μg/L) was treated with hydrocortisone (10–15 mg/m^2^/day). Central diabetes insipidus was treated with desmopressin, given orally or intranasally twice daily at a dose that kept the urine volume normal. The replacement doses did not vary with the degree of hypothalamic involvement.

Leptin and its sOB-R (both assessed with kits from Diagnostic Systems Laboratories, Inc., Webster, TX) and total ghrelin (RIA from Linco Research, St. Charles, MO) were measured on plasma samples obtained from fasting patients and stored at -20°C. All samples assayed for a given biological parameter were included in the same run. The detection limits of the assays were 0.10 μg/L for leptin, 0.14 μg/L for sOB-R, and 93 ng/L for ghrelin. Within-run coefficients of variation were less than 5% for leptin, 13% for sOB-R, and 10% for ghrelin. The sOB-R assay was not affected by adding up to 300 μg/L recombinant leptin to plasma samples. The FLI was determined by calculating the ratio between the concentrations of leptin and sOB-R, multiplied by 100. The HOMA was calculated as described in [18].

Height is expressed as SDS scores for chronological age [19], BMI as kg/m^2 ^and z-score (zs) [20], and IGF-1 as zs [21].

Data are means ± SD. The groups were compared with the Mann-Whitney U-test. The data before surgery were compared to those after surgery with the Wilcoxon signed-rank tests. Correlations were analyzed using the Spearman test.

## Results

### 1. Comparison of the groups

Despite having similar ages and BMIs, the grade 2 patients had higher glucose, insulin plasma concentrations and HOMA before surgery than did the grades 0 and 1 patients (Table 1). The grade 0 and 1 patients gained significantly less weight (kg) during the year after surgery than did the grade 2 patients (Table 2). The pre-surgery HOMA was positively correlated with these weight changes (P < 0.03).

**Table 1:** Craniopharyngioma before surgery

	**Hypothalamic involvement**					
				
Grade	**0**	**1**	**2**	0 vs 1	0 vs 2	1 vs 2
				P	P	P
Age, years	6.5 ± 4.3 (7)	8.1 ± 2.8 (8)	9.4 ± 3.1 (12)			
Height, SDS	0.46 ± 1.8 (7)	-1.1 ± 1.1 (8)	-0.57 ± 1.1 (12)	<0.04		
BMI, zs	0.20 ± 2.0 (7)	0.58 ± 1.9 (8)	1.5 ± 1.3 (12)			
Glucose, mmol/L	4.4 ± O.25 (5)	4.3 ± 0.6 (7)	5.1 ± 0.6 (10)		0.02	<0.02
Insulin, mIU/L	5.1 ± 2.4 (7)	5.4 ± 3.3 (8)	12.0 ± 11.8 (11)		<0.05	<0.03
HOMA	1.31 ± 0.6 (5)	1.28 ± 0.9 (7)	3.65 ± 3.5 (9)		0.02	<0.03
IGF-1, zs	-2.8 ± 1.7 (7)	-3.0 ± 3.1 (8)	-2.1 ± 2.2 (12)			<0.03
Leptin, μg/L	5.6 ± 2.6 (5)	7.2 ± 4.5 (8)	14.0 ± 9.8 (12)			
sOB-R, μg/L	72 ± 37 (2)	70 ± 35 (8)	50 ± 27 (7)			
Free leptin index	12.7 ± 13 (2)	12.4 ± 9.O (8)	34 ± 33 (7)			
Ghrelin, ng/L	2256 ± 331 (2)	1091 ± 251 (5)	1083 ± 222 (6)	0.05	<0.05	

mean ± SD

Number in parentheses indicate the effectives

**Table 2:** Craniopharyngioma 1 year after surgery

	**Hypothalamic involvement**					
				
Grade	**0**	**1**	**2**	0 vs 1	0 vs 2	1 vs 2
				P	P	P
Height, SDS	0.13 ± 1.3 (7)	-1.6 ± 1.1 (8)	-0.55 ± 1.3 (12)	<0.04		
BMI, zs	2.7 ± 1.1 (7)	2.5 ± 1.8 (8)	4.0 ± 1.3 (12)		<0.05	
Changes in weight, Kg	5.8 ± 4.9 (7)	7.2 ± 5.3 (8)	16.3 ± 7.4 (12)		<0.007	<0.02
Changes in weight, SDS	1.0 ± 1.2 (7)	1.2 ± 0.9 (8)	2.5 ± 1.4 (12)		<0.05	<0.04
Glucose, mmol/L	NA	3.9 ± 1.2 (2)	4.6 ± 0.7 (7)			
Insulin, mIU/L	10.1 ± 9.0 (2)	6.1 ± 3.7 (5)	47 ± 58 (10)			<0.04
HOMA	NA	0.9 ± 0.3 (2)	7.1 ± 9.0 (7)			
IGF-1, zs	-4.3 ± 0.6 (2)	-2.8 ± 2.0 (6)	-2.4 ± 0.8 (10)		<0.05	
Leptin, μg/L	14.2 ± 14 (2)	32 ± 14 (4)	61 ± 26 (10)			<0.04
sOB-R, μg/L	NA	30 ± 5.3 (4)	28 ± 7.2 (5)			
Free leptin index	NA	113 ± 63 (4)	279 ± 102 (5)			0.05
Ghrelin, ng/L	NA	994 ± 263 (4)	722 ± 126 (6)			

mean ± SD; NA: not available.

Number in parentheses indicate the effectives

The biological data were collected 6–18 months after surgery

Before surgery, the plasma IGF-1 concentrations were lower in grade 1 than in grade 2 patients, and those of ghrelin were greater in grade 0 than in grade 1 and 2 patients. After surgery, the plasma concentrations were lower in grade 0 than in grade 2 for IGF-1, and in grade 1 than in grade 2 for insulin and leptin.

### 2. Comparison of the variables in the whole population

**Figure 1:**
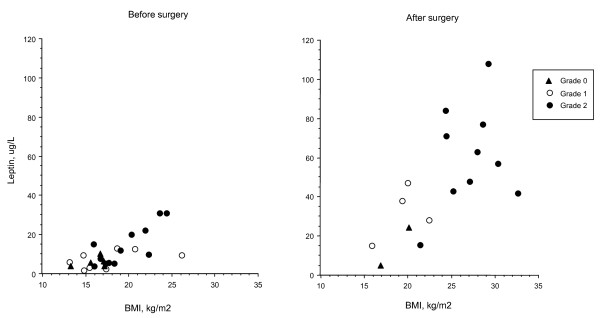
**Comparison of leptin with BMI (kg/m^2^) before (n = 25) and after (n = 16) surgery for craniopharyngioma according to the hypothalamic involvement**.

The data for the whole population before and 6–18 months after surgery showed increases in BMI (P < 0.0001), insulin (P < 0.005), leptin (P = 0.0005), FLI (P < 0.008), and decreases in sOB-R (P < 0.04) and ghrelin (P < 0.03). The comparison of leptin with BMI before and after surgery according to the hypothalamic involvement is shown on Figure 1.

The BMIs before and after surgery were positively correlated with the HOMA and with the plasma concentrations of insulin and leptin, as were the insulin values with IGF-1 (Table 3).

**Table 3:** Comparison of the variables in the whole population of craniopharyngioma

	**Before surgery**		**After surgery**		**Before and after**	
			
	Rho	P	Rho	P	Rho	P
BMI (zs) vs insulin	0.49	<0.02			0.54	<0.0006
BMI (kg/m2) vs insulin	0.53	<0.008	0.79	<0.003	0.66	<0.0001
BMI (zs) vs HOMA					0.41	<0.03
BMI (kg/m2) vs HOMA	0.50	<0.03	0.78	<0.03	0.58	<0.002
BMI (zs) vs IGF-1			0.51	<0.04		
BMI (kg/m2) vs IGF-1			0.71	<0.006	0.35	<0.03
BMI (zs) vs leptin	0.65	<0.002	0.57	<0.03	0.80	<0.0001
BMI (kg/m2) vs leptin	0.62	<0.003	0.67	<0.01	0.80	<0.0001
BMI (zs) vs sOB-R					-0.54	<0.007
BMI (kg/m2) vs sOB-R					-0.50	<0.02
BMI (zs) vs FLI	0.55	<0.03			0.77	0.0001
BMI (kg/m2) vs FLI					0.73	0.0003
BMI (zs) vs ghrelin					-0.54	<0.02
BMI (kg/m2) vs ghrelin					-0.56	<0.01
Insulin vs delta kg						
Insulin vs IGF-1	0.48	<0.02	0.69	<0.008	0.54	0.0005
Insulin vs leptin			0.55	<0.04	0.56	0.0005
Insulin vs sOB-R						
Insulin vs ghrelin			-0.71	<0.04	-0.52	<0.02
IGF-1 vs leptin			0.74	<0.005		
IGF-1 vs sOB-R						
IGF-1 vs ghrelin			-0.74	<0.03		
Leptin vs sOB-R					-0.69	0.0005
Leptin vs ghrelin			-0.67	<0.05	-0.63	<0.004

After surgery, the plasma concentrations of leptin were positively correlated with those of insulin and IGF-1. The plasma concentrations of ghrelin were negatively correlated with those of insulin, IGF-1 and leptin.

When the data before and after surgery were analysed together, the leptin concentrations were negatively correlated with those of sOB-R.

## Discussion

Our main finding is that the hypothalamic involvement by the craniopharyngioma before surgery seems to determine the degree of insulin resistance evaluated by the HOMA, regardless of the BMI. In turn, the pre-surgery HOMA values were correlated with the post-surgery weight gain.

### 1. Hypothalamic involvement

We find that a greater hypothalamic involvement before surgery, as evaluated by MRI, is associated with significantly higher glucose and insulin plasma concentrations and HOMA, and a lower concentration of ghrelin. There was no significant association between the BMI and the degree of hypothalamic involvement in the whole population or in the 21/27 patients for whom the HOMA was available. This suggests that the insulin resistance before surgery was mainly due to the hypothalamic damage caused by the craniopharyngioma. One grade 2 patient with a high HOMA before surgery (2.93) developed diabetes at 22.4 years, with high fasting plasma insulin concentrations (27 mIU/L); treatment with Metformine and Glimepiride normalised the blood glucose concentration.

The changes in weight during the year after surgery differed according to the hypothalamic involvement. The positive correlation between the HOMA before surgery and these changes suggests that the insulin resistance induced by the craniopharyngioma itself influences the body weight after surgery.

De Vile et al [9] used MRI to classify the hypothalamic involvement in children with craniopharyngioma 1.2–19.2 years after surgery. They showed that the 10/17 patients with the greatest involvement had a history of extreme weight loss or weight gain at presentation, and a significantly greater increase in BMI at follow-up than those who had no, or intermediate hypothalamic involvement. Müller et al [11,12] showed that hypothalamic involvement and a familial disposition to obesity seem to have a major impact on the development of obesity, whereas endocrine deficiencies and hormone replacement therapy do not.

### 2. Mechanism of the obesity

It has been suggested that the craniopharyngioma and/or surgery disturb the feedback control of leptin secretion. Brabant et al [14] found that the plasma leptin concentrations of patients with a pituitary adenoma were comparable to those of controls, whereas 7/18 patients with craniopharyngioma had higher than expected leptin concentrations for their BMI. This was also reported for 11 patients with a suprasellar craniopharyngioma, whereas 3 patients with intrasellar craniopharyngioma had lower, almost normal leptin concentrations [15]. In contrast, Srinivasan et al [22] reported equally high leptin concentrations in children after removal of the craniopharyngioma and in controls matched for their BMI. They also found no difference in total body fat or in the reduction in insulin sensitivity. We [23] have shown that the obesity that follows cachexia after the treatment of hypothalamic tumors in children younger than one year old is not due to dysregulation of leptin secretion, and that the leptin concentrations and sOB-R remain regulated by factors like testosterone. The sOB-R decreased in all but one patient from before to after surgery. This decrease was significant and concomitant with the increases in BMI and leptin. The sOB-R concentrations were negatively correlated with the BMI and the leptin concentrations when the data before and after surgery were analysed together. This suggests that the relationship between leptin and its receptor is not altered by the surgery.

Goldstone et al [16] showed that the fasting plasma ghrelin concentrations of adults operated on for craniopharyngioma were negatively correlated with the percent of body fat, plasma insulin and HOMA. We find that the plasma ghrelin concentrations before surgery are not correlated with the other variables. They decreased significantly after surgery to become closely and negatively correlated with the concentrations of insulin (with a correlation coefficient similar to that found by Goldstone), IGF-1 and leptin.

The plasma IGF-1 concentrations were positively correlated with insulin before surgery, and with BMI, insulin and leptin after surgery. They were greater in the group with hypothalamic involvement than in the group without such involvement. They were over -2 zs in 3/26 patients, despite the complete GH deficiency. This suggests that insulin stimulates IGF-1 production, and may explain the normal growth rate of these patients despite their GH deficiency.

### 3. Forces, limitations and medical implications

All patients had craniopharyngioma and were evaluated before and after surgery. The second evaluation was performed before GH or sex steroid treatment was started. They were all operated on in the same department and followed by the same endocrinologist. But not all the parameters were assayed in each sample.

There is, presently, no effective long-term treatment for these patients. Lustig et al [5,6] treated patients with hypothalamic obesity with somatostatin agonist. This promoted weight loss, which was correlated with a reduction in insulin secretion on oral glucose tolerance testing and in leptin concentrations. Srinivasan et al [22] found no differences in the metabolic parameters and body compositions of 5 craniopharyngioma cases treated with GH and 10 untreated cases. Geffner et al [24] showed that treating children with craniopharyngioma with GH for 3 years did not modulate the weight gain. Mason et al [25] treated with dextroamphetamine 5 patients with significant weight gain and poor attention following surgical treatment of craniopharyngioma, and found an improvement. Alemzadeh et al [26] found that diazoxide (2 mg/kg/day in 3 doses p.o.) decreased the weight and plasma insulin of obese hyperinsulinemic adults.

## Conclusion

The degree of hypothalamic involvement determines the insulin secretion, and possibly that of ghrelin, which determine the BMI and its evolution; this, in turn determines the leptin concentration. Thus, obesity should be prevented by reducing insulin secretion in those cases with hypothalamic involvement.

## Abbreviations

BMI: body mass index; GH: growth hormone; FLI: free leptin index; HOMA: homeostasis model insulin resistance index; IGF: insulin-like growth factor; MRI: magnetic resonance imaging; sOB-R: soluble leptin receptor; zs: z-score.

## Competing interests

The authors declare that they have no competing interests.

## Authors' contributions

CT and JCS carried out the immunoassays and performed the statistical analyses. KB, NM and CR participated in the conception and design, data acquisition and analysis. MZ and CSR operated on the patients. CSR reviewed the neuroradiological data. RB directed the work and prepared the manuscript. All the authors have given final approval of the version to be published.

## Pre-publication history

The pre-publication history for this paper can be accessed here:


